# Impaired Interhemispheric Synchrony in Parkinson’s Disease with Fatigue

**DOI:** 10.3390/jpm12060884

**Published:** 2022-05-27

**Authors:** Yong-Sheng Yuan, Min Ji, Cai-Ting Gan, Hui-Min Sun, Li-Na Wang, Ke-Zhong Zhang

**Affiliations:** Department of Neurology, The First Affiliated Hospital of Nanjing Medical University, Nanjing 210029, China; da_sheng@126.com (Y.-S.Y.); 18351998901@163.com (M.J.); gct18351975877@126.com (C.-T.G.); m18021072582@163.com (H.-M.S.); 15895901868@126.com (L.-N.W.)

**Keywords:** Parkinson’s disease, fatigue, functional magnetic resonance imaging, voxel-mirrored homotopic connectivity, supramarginal gyri

## Abstract

The characteristics of interhemispheric resting-state functional connectivity (FC) in Parkinson’s disease (PD) with fatigue remain unclear; therefore, we aimed to explore the changes in interhemispheric FC in PD patients with fatigue. Sixteen PD patients with fatigue (PDF), 16 PD patients without fatigue (PDNF) and 15 matched healthy controls (HCs) were enrolled in the retrospective cross-sectional study. We used voxel-mirrored homotopic connectivity (VMHC) to analyze the resting-state functional magnetic resonance imaging (fMRI) data of these subjects. Compared to PDNF, PDF patients had decreased VMHC values in the supramarginal gyri (SMG). Furthermore, the mean VMHC values of the SMG were negatively correlated with the mean fatigue severity scale (FSS/9) scores (*r* = −0.754, *p* = 0.001). Compared to HCs, PDF patients had decreased VMHC in the SMG and in the opercular parts of the inferior frontal gyri (IFG operc). The VMHC values in the IFG operc and middle frontal gyri (MFG) were notably decreased in PDNF patients compared with HCs. Our findings suggest that the reduced VMHC values within the bilateral SMG may be the unique imaging features of fatigue in PD, and may illuminate the neural mechanisms of fatigue in PD.

## 1. Introduction

Fatigue is one of the most frequent non-motor symptoms of Parkinson’s disease (PD), which is described as a significantly diminished energy level or an increased perception of effort that is disproportionate to the attempted activities. It affects about 33–58% of PD patients [1,2,3] and impairs quality of life [4]. While the pathophysiology of fatigue in PD patients is not clear, many studies have attempted to probe the potential mechanisms within the last decade. An early study found a correlation between fatigue and frontal lobe hypoperfusion in PD patients [5]. Subsequently, neuroimaging studies demonstrated abnormal regional cerebral blood flow (rCBF) and glucose metabolism in the frontal lobe, caudate, insula, middle temporal gyrus, precuneus and middle occipital gyrus in PD-related fatigue [6,7]. Furthermore, functional magnetic resonance imaging (fMRI) studies reported abnormal local activities in cognitive regions, including the left anterior cingulate cortex (ACC), right superior frontal gyrus (dorsolateral part), left postcentral gyrus and right inferior frontal gyrus (orbital and triangular part) for chronic fatigue [8,9], which may have similar pathological mechanisms with fatigue in PD. Thereafter, Cho et al. considered that impaired activation of the salience network, which mainly comprises bilateral anterior insulas and the anterior cingulate cortex, could lead to a persistent broad and unfocused mental state, resulting in distracting, internally focused information, which could contribute to fatigue in PD patients [10]. Recently, a group-level independent component analysis demonstrated the increased connectivity of DMN in PD patients with fatigue, which may suggest that a higher attention level could represent an initial cognitive compensatory response, as a manifestation of cognitive cortical plasticity [11]. These prior outcomes suggest that fatigue is associated with the dysfunctional regions implicated in cognition function, such as the regulation of attention.

However, little research pointed to the changes in functional connectivity between two hemispheres. In fact, PD is clinically manifested as unilateral onset, and is asymmetrical during the progression of the disease on both sides of the body, indicating the discordancy in the pathological damage to the two hemispheres [12,13]. Meanwhile, previous studies revealed that PD patients with fatigue had abnormal motor and cognitive network connectivity [14], which was mainly regulated by the right hemisphere due to its dominance for attention and arousal [15]. So, the laterality of cognitive networks might have an impact on fatigue in PD. Therefore, it would be meaningful to pay close attention to the interhemispheric resting-state FC in fatigue, which can identify the characteristics of intrinsic functional architecture between geometrically corresponding regions in each hemisphere and reflect interhemispheric communication to the integrated brain function underlying coherent cognition and behavior [16]. Voxel-mirrored homotopic connectivity (VMHC) is a method of measuring the interhemispheric resting-state FC [16]. It has not been thoroughly applied to evaluate the neuroimaging features of fatigue in PD patients. Here, we explored the hypothesis that PD patients with fatigue (PDF) would exhibit abnormal interhemispheric FC in the brain regions associated with cognition, compared with non-fatigued PD patients (PDNF) and healthy controls (HCs) by VMHC. We expected that the cognitive regions would be distinctively influenced in PDF patients, except for motor-related areas, which would be affected in both PDF and PDNF patients.

## 2. Materials and Methods

### 2.1. Participants and Clinical Assessment

A cross-sectional retrospective study was designed. PD patients were consecutively recruited from the Department of Neurology in the First Affiliated Hospital of Nanjing Medical University between March 2018 and April 2020. Meanwhile, healthy controls (HCs) were consecutively recruited from hospital personnel and society. This study conformed to the standards set by the latest revised version of the Declaration of Helsinki (as revised in 2013) and was approved by the ethics committee of the First Affiliated Hospital of Nanjing Medical University. All participants signed informed consent before beginning the experiment.

**Figure 1:**
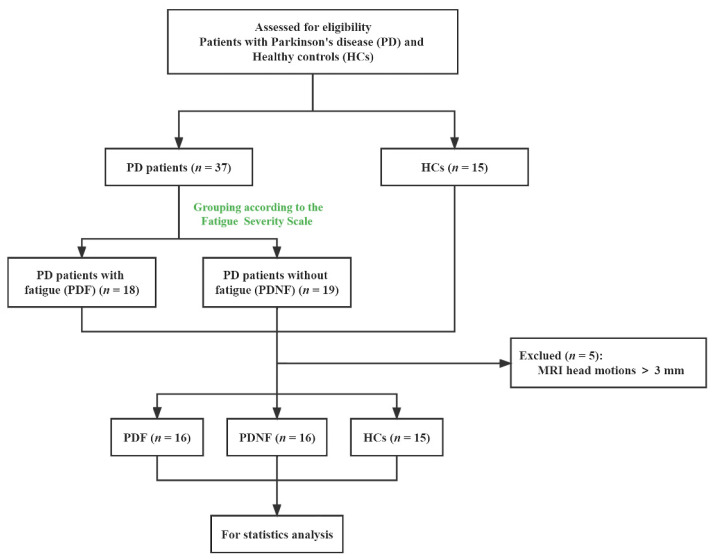
Study flow diagram. MRI: magnetic resonance imaging.

The diagnosis of definite PD met the criteria of the British Parkinson’s Disease Society Brain Bank for PD [17]. The exclusion criteria were as follows: (1) uncertain diagnosis of PD or parkinsonian plus syndromes; (2) diagnosis of severe neurological or psychiatric diseases; (3) contraindications for MRI scan; (4) taking antidepressants or medications that have fatigue as a side effect according to the package insert, or having other diseases that can result in the onset of fatigue; (5) other confounding factors associated with fatigue, such as significant cognitive dysfunction (Mini-Mental State Examination (MMSE) scores < 24), moderate or severe depression (the 24-item Hamilton Depression Rating Scale (HAMD) > 17), apathy (apathy scale (AS) > 14) and excessive daytime sleepiness (Epworth sleepiness scale (ESS) > 10). Finally, 37 idiopathic PD patients and 15 HCs matched with age, sex and education were enrolled and all subjects were right-handed. However, the data of only 32 PD patients and 15 HCs were analyzed because 5 patients (2 fatigued patients and 3 non-fatigued patients) were excluded due to abnormal head motions. The flow chart is shown in Figure 1.

All subjects underwent the scale evaluation and MRI examinations after more than 12 h withdrawal from antiparkinsonian medications to alleviate the pharmacological effects on neural activity. The presence and severity of fatigue were defined by the fatigue severity scale (FSS), which has 9 items and was widely used in PD, owing to its reliability, validity and sensitivity for detection of fatigue symptoms [18]. Patients were divided into two groups based on the presence (*n* = 18) or absence (*n* = 19) of fatigue. PD patients with a mean FSS (FSS/9) score > 4.0 were assigned to the PDF group, while the remaining patients were enrolled in the PDNF group. In addition, the disease duration, severity and non-motor symptoms were evaluated by clinical scales, including Hoehn and Yahr (H&Y) stage, the motor element of Unified Parkinson’s Disease Rating Scale (UPDRS-III), MMSE, ESS, AS, HAMD, and HAMA (Hamilton Anxiety Rating Scale). According to recognized methods [19], we calculated the levodopa equivalent daily dose (LEDD) for each patient.

### 2.2. Image Acquisition

MRI data from all subjects were obtained using a Siemens 3.0-Tesla signal scanner (Siemens Medical Solutions, Erlangen, Germany). In order to reduce scanner noise and limit head motions, participants were fitted with foam padding and earplugs, then were instructed to close their eyes, remain still, stay awake, and not think of anything during scanning. High-resolution brain structural images were obtained using T1-weighted, sagittal 3D magnetization-prepared rapid gradient echo (MPRAGE) sequences with the following parameters: repetition time (TR) = 1900 ms, echo time (TE) = 2.95 ms, flip angle (FA) = 9^°^, slice thickness = 1 mm, slices = 160, field of view (FOV) = 230 × 230 mm^2^, matrix size = 256 × 256, and voxel size = 1 × 1 × 1 mm^3^. Functional images were acquired using an echo-planar imaging (EPI) sequence (TR = 2000 ms, TE = 21 ms, FA = 90°, FOV = 256 × 256 mm^2^, in-plane matrix = 64 × 64, slices = 35, slice thickness = 3 mm, no slice gap, voxel size = 3 × 3 × 3 mm^3^, and total volumes = 240) on each subject.

### 2.3. Data Preprocessing

Rs-fMRI data preprocessing was executed on Data Processing Assistant for Resting-State fMRI (DPARSF, http://www.restfmri.net/forum/dparsf, accessed on 10 November 2021). With reference to this literature [20], we partitioned the preprocessing strategies into the following steps. To begin with, the first 10 time points were disposed of and the remaining 230 images were revised for timing differences between slices and head motion (Friston 24 parameter), taking the middle layer as the reference slice. Subsequently, individual T1 structural images were co-registered to the mean EPI scans and segmented into gray matter and white matter by “New Segment”. Then, the transformations were computed from the native space to the Montreal Neurological Institute (MNI) space by DARTEL normalization and applied to spatially normalize the EPI images. The following steps were implemented: resampling with 3 × 3 × 3 mm^3^ resolution, spatially smoothing with a 6 mm full-width half-maximum Gaussian kernel to decrease spatial noise, removing the linear trend and temporally filtering (0.01–0.08 Hz). Several sources of spurious variance were regressed out, including the white matter signal, the cerebral spinal fluid signal, and six head motion parameters obtained by head motion correction. Five participants (2 fatigued patients and 3 non-fatigued patients) with head motions more than 3.0 mm of translation or 3.0° of rotation were excluded.

### 2.4. Voxel-Mirrored Homotopic Connectivity

The values of VMHC were calculated with REST software (http://restfmri.net, accessed on 10 November 2021), and on the basis of the Gan’s article [20]. First, a mean normalized T1 image was established by averaging the spatially normalized T1 images. Afterwards, a group-specific symmetric brain template was created by averaging the above resulting T1 image with its left-right mirrored version, which was used for nonlinear registration of the individual T1 images. The identical transformation was applied to the resting fMRI images. For each subject, the Pearson correlation coefficient was computed between any pair of symmetric interhemispheric voxels and correlation values were then Fisher z-transformed to improve the normality. The resultant values constituted the VMHC and were used for the group analysis.

### 2.5. Statistical Analysis

Values of demographic and clinical variables were expressed as the mean ± standard deviation (SD). All analyses were processed by SPSS 21.0 (SPSS Inc, Chicago, IL, USA). One-way analysis of variance (ANOVA), Kruskal–Wallis test, the Pearson χ^2^ test, Student’s t test, and Mann–Whitney U test were used to analyze the differences among the three groups (PDF, PDNF and HCs) regarding demographic and clinical variables, then least significant difference(LSD) was used for post hoc tests, as appropriate. *p* < 0.05 (two-tailed) was considered statistically significant.

Voxel-based comparisons of the entire VMHC maps were conducted with REST software (http://restfmri.net, accessed on 10 November 2021). Statistical tests were as follows: first, the one-way analysis of covariance (ANCOVA) to identify brain areas with significant differences in VMHC among the three groups with age, sex, and education level as covariates, followed by post hoc two-sample t tests. The ANCOVA result was corrected by AlphaSim correction (http://afni.nimh.nih.gov/pub/dist/doc/manual/AlphaSim.pdf, accessed on 10 November 2021), with a voxel-level *p* < 0.01 and cluster size > 58 voxels, corresponding to a corrected *p* < 0.01. The post hoc two-sample t tests were conducted within a mask showing significant differences obtained from the ANCOVA analysis, with corrections (voxel-level *p* < 0.01; cluster size > 10 voxels, determined by a Monte Carlo simulation that resulted in a cluster-level significance threshold of *p* < 0.01).

The brain areas showing significant differences between PDF and PDNF patients were selected as regions of interest (ROIs). Afterwards, Pearson correlation coefficients were computed between the extracted mean VMHC values within the ROIs and the FSS/9 scores of PDF patients. The significance level was set at *p* < 0.05 (two-tailed).

## 3. Results

### 3.1. Demographic and Clinical Characteristics

The demographic and clinical data are summarized in Table 1. There were no significant differences in age, sex, education levels and MMSE among the three groups. Similarly, no significant differences were detected for PDF and PDNF groups, in terms of disease duration, H&Y stage, UPDRS-III, LEDD, ESS, AS, HAMD and HAMA. As expected, the FSS/9 value was significantly higher in the PDF group than the PDNF group (mean difference = 3.06, 95% confidence interval [CI] 2.52 to 3.83, *p* < 0.001).

### 3.2. Voxel-Mirrored Homotopic Connectivity

**Figure 2:**
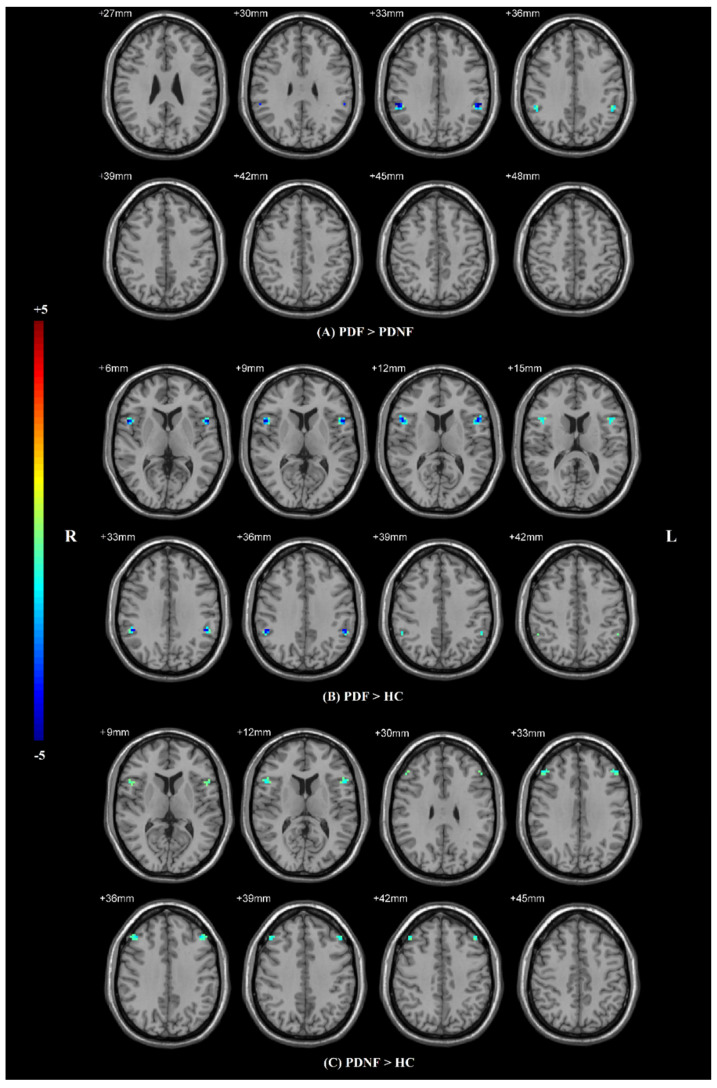
Statistical maps showing VMHC differences in different brain regions between three groups. The results were corrected by AlphaSim (with a combined threshold of *p* < 0.01). (**A**) Differences between PDF patients and PDNF patients; (**B**) differences between PDF patients and HC group; (**C**) differences between PDNF patients and HC group. Abbreviations: VMHC: voxel-mirrored homotopic connectivity, HCs: healthy controls, PDF: Parkinson’s disease with fatigue, PDNF: Parkinson’s disease without fatigue, L: left, R: right.

ANCOVA revealed significant differences in VMHC among PDF, PDNF and HC groups, where age, sex, and education level were included as covariates, followed by a post hoc t-test within the mask obtained from the ANCOVA analysis. Compared to the PDNF group, PDF had decreased VMHC values in the supramarginal gyri (SMG) (*p* < 0.01) (Table 2; Figure 2A). Compared to HCs, PDF had lower VMHC values in the SMG (*p* = 0.005) and the opercular parts of inferior frontal gyri (IFG operc) (*p* < 0.01) (Table 2; Figure 2B). Meanwhile, VMHC in the IFG operc (*p* = 0.001) and middle frontal gyri (MFG) (*p* < 0.01) were notably decreased in PDNF patients compared with HCs (Table 2; Figure 2C).

### 3.3. Correlation Analysis

**Figure 3:**
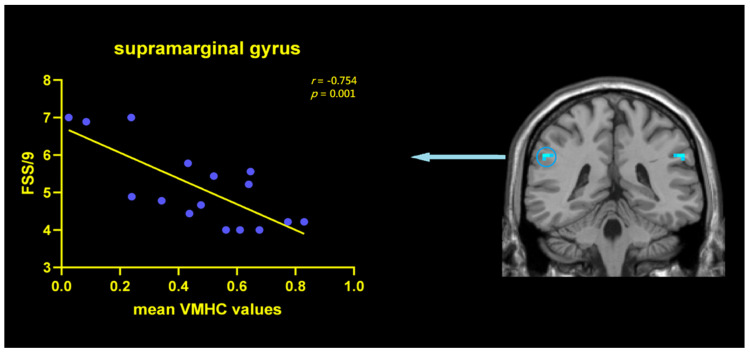
Correlations between VMHC values in SMG and FSS/9 scores within the PDF patients. Scatterplots demonstrated that there was a significant negative correlation between the mean VMHC values in the SMG (r = −0.754, *p* = 0.001) and FSS/9 scores in PDF patients. Abbreviations: VMHC: voxel-mirrored homotopic connectivity; FSS: fatigue severity scale; SMG: supramarginal gyrus; PDF: Parkinson’s disease with fatigue.

Based on the VMHC results, correlation analyses between FSS/9 scores and mean VMHC values of SMG were conducted for PDF patients. FSS/9 scores were negatively correlated with the mean VMHC signals within the SMG regions (*r* = −0.754, *p* = 0.001) (Figure 3), indicating that with the aggravation of fatigue, the function coordination of SMG would be poorer in PDF patients. However, this correlation was not found in the PDNF group (*r* = −0.155, *p* = 0.566).

## 4. Discussion

Based on the clinical characteristics of the unilateral onset of PD and the asymmetry of functional connectivity and structural changes in the bilateral hemispheres in PD with fatigue [13,14,21,22,23,24], we applied VMHC, which can detect interhemispheric functional asynchronization sensitively, to detect functional coordination between hemispheres in fatigue. Moreover, this study represented the first attempt to characterize fatigue-related interhemispheric brain synchrony abnormalities in patients with PD by the VMHC methodology. Compared to both PDNF and HC groups, we found that the PDF group had lower VMHC values in the bilateral SMG, which makes valuable contributions to cognitive function, such as attentional control and emotional regulation [25]. Additionally, when compared to the HCs, the PDF and PDNF patients both showed reduced VMHC values, mainly in the bilateral IFG operc, and PDNF patients displayed an additional decrease in the VMHC values of bilateral MFG. Furthermore, a significant negative correlation was found between FSS/9 scores and the VMHC values of the SMG in the PDF patients, indicating that the decreased VMHC values of SMG might be associated with the severity of PD-related fatigue.

The SMG plays an important role in cognitive circuits [26], which is mainly reflected in bottom-up attention [27], motor control [28] and emotional modulation, especially the down-regulation of negative emotion [29]. The latest study found that the right SMG was one of the fronto-parietal connector hubs, interacting functionally with the dorsal attention network and the ventral attention network [30], which showed differences in the processing of attention information in the bilateral SMG. Meanwhile, fatigue complaints were associated with cognitive impairment in a large PD cohort [31]. Pavese N. et al. found that the feeling of fatigue was often caused by the dissociation of motivation from executive motor movement [6], which depended on the mechanisms of evaluating and comparing the costs and benefits of a motor/mental activity by balancing its energetic cost with the volitional and motivational drive of subjects [32]. Moreover, two studies demonstrated that fatigue was related to the inability to process external stimuli correctly, and PDF patients had difficulty in attentional orienting to salient novel stimuli, which correlated with the severity of subjective fatigue [27,33]. Additionally, recent resting-state fMRI studies found that PD-related fatigue was related to altered neural activity in the areas implicated in the attention, salience and default networks [11,34]. Abnormal functional connectivity in the parietal lobe containing SMG was disclosed in chronic fatigue syndrome [8]. According to some perspectives, fatigue in PD is essentially a cognitive impairment, mainly manifested by abnormal regulation of the attention domain [6]. Particularly, SMG is an important part of this loop. Further correlation analyses showed that the decreased VMHC values in SMG were only associated with FSS/9 scores in the PDF patients, indicating that the decreased VMHC values in SMG were specific in PD patients with fatigue. Hence, we speculated that the reduced VMHC within the bilateral SMG may possibly underlie the neural mechanisms of fatigue in PD, by affecting the synergistic function of bilateral attentional regulation related to cognitive function, and such dysfunction could be accompanied by micro injury of white matter tracts in the cerebral hemisphere [35]. Furthermore, we surmised that SMG might be a possible target for neuromodulation strategies (e.g., implemented by transcranial magnetic stimulation or transcranial direct current stimulation techniques) by modulating SMG activity to ameliorate PD fatigue. In the future, rigorous clinical trials are needed to verify our hypothesis.

Additionally, the comparison between the PD patients (PDF or PDNF) and the HC group revealed reduced interhemispheric synchrony within the bilateral IFG operc. Generally, the cardinal symptoms of PD are thought to be attributed to the dysfunction of motor circuits [36,37] and sensory processing [28]. As we know, the IFG operc is a crucial component of the cognitive locomotor control and self-regulation network [38] involved in corticocortical and subcortical pathways during motor and cognitive inhibition [39,40]. Thus, we supposed that the reduced interhemispheric FC within IFG operc might indicate poor coordination of the two hemispheres in motor control and sensory perception in PD, simultaneously. In agreement with previous studies [41,42], we also observed that, compared with HCs, PDNF patients showed decreased VMHC values in the MFG, known as a key node for regulating motor and non-motor symptoms [43].

The present study has several limitations besides the small sample size. First, the use of drugs could be an important confounding factor. However, we evaluated all patients during off state and the doses of dopaminergic drug usage were well matched in both the PDF and PDNF groups. Second, the brain is not exactly structurally symmetrical. Thus, we smoothed the functional data and normalized them to a symmetric template for resolving this issue as much as possible [16]. Third, the sample size of our PD patients could limit the generalizability of the current results, and further research is needed to verify whether the present results can be applied to individuals, groups or populations with different ages and severities of disease.

## 5. Conclusions

To sum up, the decreased VMHC values within the bilateral SMG may be the unique imaging features of fatigue in PD. This discovery also suggests that the uncoordinated function of bilateral SMG may have participated in the pathophysiological mechanisms of fatigue in PD, possibly via affecting cognitive function involving attention. We hope that this study can provide some theoretical accumulation for the diagnosis and clinical treatment of fatigue in PD patients.

## Figures and Tables

**Table 1:** Demographic and clinical characteristics of all subjects.

Variables	PDF (*n* = 16)	PDNF (*n* = 16)	HCs (*n* = 15)	*p* Value
Age (y) ^a^	57.25 ± 13.98	63.37 ± 9.19	63.80 ± 5.72	0.147
Sex (F/M) ^b^	8/8	4/12	5/10	0.326
Education (y) ^c^	11.68 ± 3.43	11.06 ± 4.15	11.33 ± 3.45	0.922
MMSE ^c^	28.25 ± 1.34	28.43 ± 1.20	28.93 ± 1.16	0.317
Disease duration (y) ^d^	5.37 ± 3.52	6.50 ± 3.38	NA	0.296
H&Y ^d^	2.34 ± 0.67	2.00 ± 0.60	NA	0.130
UPDRS-III ^d^	29.00 ± 11.00	28.31 ± 11.72	NA	0.865
LEDD (mg/day) ^d^	613.67 ± 248.89	659.68 ± 349.92	NA	0.671
ESS ^d^	5.06 ± 3.67	4.06 ± 3.21	NA	0.419
AS ^d^	10.06 ± 2.69	8.31 ± 3.51	NA	0.125
HAMD ^e^	10.38 ± 4.98	7.94 ± 4.16	NA	0.143
HAMA ^e^	10.56 ± 4.75	8.44 ± 5.42	NA	0.247
FSS/9 ^a^	5.13 ± 1.06	2.07 ± 1.06	1.50 ± 0.42	<0.001 *
Post hoc	PDF vs. PDNF			<0.001 *
	PDF vs. HC			<0.001 *
	PDNF vs. HC			0.089

Values are presented as the mean ± standard deviation. ^a^ One-way ANOVA test, ^b^ chi-square test, ^c^ Kruskal–Wallis test, ^d^ Mann–Whitney U test, ^e^ Student’s t test. * *p* < 0.05 was considered significant. Abbreviations: HCs: healthy controls, PDF: Parkinson’s disease with fatigue, PDNF: Parkinson’s disease without fatigue, NA: not applicable, F: Female, M: Male, y: year, MMSE: Mini-Mental State Examination, H&Y: Hoehn and Yahr stage, UPDRS: unified Parkinson’s disease rating scale, LEDD: levodopa equivalent daily dose, AS: apathy scale, ESS: Epworth Sleepiness Scale, HAMD: 24-item Hamilton Depression Rating Scale, HAMA: Hamilton Anxiety Rating Scale, FSS: fatigue severity scale.

**Table 2:** Regions showing significant differences in VMHC between groups.

Brain Regions (AAL)	Number of Voxels	MNI Coordinates			*T* Value
		X	Y	Z	
PDF > PDNF					
SMG	15	±54	−39	33	−3.9081
PDF > HCs					
IFG operc	36	±54	12	6	−4.7763
SMG	15	±51	−42	36	−4.4129
PDNF > HCs					
IFG operc	12	±54	15	12	−3.6089
MFG	27	±42	33	36	−3.8621

A corrected threshold of *p* < 0.01, corrected by Monte Carlo. Anatomical Automatic Labeling 90 was used to report the anatomical regions of our clusters. Abbreviations: HCs: healthy controls, PDF: Parkinson’s disease with fatigue, PDNF: Parkinson’s disease without fatigue, MNI: Montreal Neurological Institute, SMG: supramarginal gyri, IFG operc: opercular parts of inferior frontal gyri, MFG: middle frontal gyri.

## Data Availability

The data supporting reported results are available from the corresponding author upon reasonable request. Computer Software for VMHC analysis were the Data Processing Assistant for Resting-State fMRI (DPARSF, http://www.restfmri.net/forum/dparsf, accessed on 10 November 2021) and REST (http://restfmri.net, accessed on 10 November 2021).
